# Promoting physician-patient language concordance in undergraduate medical education: a peer assisted learning approach

**DOI:** 10.1186/s12909-022-03986-4

**Published:** 2023-01-03

**Authors:** Sawsan Ismaiel, Dana AlGhafari, Halah Ibrahim

**Affiliations:** Khalifa University College of Medicine and Health Sciences, PO Box 127788, Abu Dhabi, United Arab Emirates

**Keywords:** Peer Assisted Learning (PAL), Medical students, Arabic language, Learning communities, Language barriers, Limited English proficiency, Medical terminology, Foreign language

## Abstract

**Background:**

Verbal communication plays an important role in the patient-physician relationship. Research shows that language concordance, when a healthcare professional communicates fluently in the patient’s preferred language, contributes to patient satisfaction and improves healthcare outcomes. Yet, many medical schools worldwide, including most institutions in the Arab world, use English as the language of instruction. As a result, students lack confidence and feel unprepared to communicate effectively with the local population. This manuscript describes the development, implementation and early perceptions of an Arabic language program for medical students in the United Arab Emirates.

**Methods:**

In 2020, the learning communities at Khalifa University College of Medicine and Health Sciences launched a pilot program implementing a Peer Assisted Learning (PAL) framework to teach Arabic medical terminology and language to both native and non-native Arabic speaking medical students. A web-based survey was administered to the first two cohorts of students to assess satisfaction with the classes and the program’s impact on students’ communication skills during clinical encounters.

**Results:**

Early perceptions of the program were very positive, with 43/48 students (89.6%) reporting that they used the information during home visits and clinical rotations, and 42 students (87.5%) admitting that the classes made them feel more comfortable in communicating with the Arabic speaking local patient population.

**Conclusion:**

This paper explores a new educational approach to address the challenge of language barriers in healthcare. A feasible, low cost program using peer assisted learning can improve students’ comfort in communicating with patients in the local language.

**Supplementary Information:**

The online version contains supplementary material available at 10.1186/s12909-022-03986-4.

## Background

As global healthcare systems move toward patient-centered and diversity-competent care, there is an increased emphasis on physician-patient communication. Verbal communication plays an important role in establishing an emotional connection between patients and physicians [1]. Commonalities and synchrony in language style express empathy, create feelings of emotional closeness, and help facilitate information sharing between patient and physician [1, 2]. Therefore, attention to language concordance, when a healthcare professional communicates fluently in the patient’s preferred language, is important in promoting high quality patient care. Research demonstrates that language concordance is positively correlated with access to care, medication adherence, and perceived quality of care [3]. Patients with language-concordant physicians are more engaged in treatment plans, participate more in screening and preventive services, and have better health outcomes, including improved blood pressure and glycemic control [4]. Moreover, language concordance can facilitate reciprocal trust to help build emotionally satisfying relationships for both patients and physicians [1, 5].

Yet, medical education systems in many countries are not designed to prioritize language concordance with the local community. English has been adopted as the preferred medium of instruction in medical education worldwide, even in countries where the language of the majority of the population is not English [6]. One study showed that 37.5% of medical schools outside of Canada and the United States use English as the medium of instruction, although English is an official language in only 22% of these countries [7]. In the Arab world, only Syria uses Arabic as the language of instruction in all of its medical schools. Medical schools in Tunisia, Morocco, Algeria, and Mauritania teach in French, while English is the primary medium of instruction throughout the remaining 17 countries [8]. The preference for English as the language for medical education aligns with the broader adoption of English by the international medical community for most medical resources, including textbooks, journals, international examinations, conferences, and continuing professional development activities [9]. Further, in an increasingly globalized and interconnected world, medical students who seek specialty training or future career opportunities abroad need to be proficient in English.

Recent studies have raised concerns about the impact of studying medicine in a foreign language on a physician’s ability to effectively communicate with and engage the local community. In India, medical students who were taught in English reported difficulties communicating with patients in local dialects [10]. Similarly, students from an English curriculum medical school in South Africa struggled to respond to the needs of the isiZulu community in which they worked [11]. There is growing evidence that medical students in the Arab world are facing similar challenges. In a study of 300 medical students in Egypt, 62% felt that studying in English created a gap between their education and clinical practice [12]. Surveys of medical students in Saudi Arabia and the United Arab Emirates (UAE) revealed that despite being native Arabic speakers, only 34% and 28%, respectively, felt confident performing a medical history in Arabic [13, 14]. Another study in the UAE showed that students, who were taught in English, had difficulty eliciting patients’ feelings and expressing empathy [15]. To address these concerns, we developed a longitudinal Arabic medical terminology and language program, offered to both native and non-native Arabic speakers in an English curriculum medical school in the UAE. The purpose of this manuscript is to describe the structure and implementation of this program, and to assess its impact on students’ comfort and perceived competence in communicating with the local patient population.

## Methods

### Setting and participants

Khalifa University College of Medicine and Health Sciences (KU CMHS) is a new medical school in the UAE. Since its inaugural class in 2019, KU CMHS has offered a United States model of medical education to students from all over the world [16]. All courses, clinical skills sessions, and Objective Structured Clinical Examinations (OSCE) are conducted in English, though the predominantly spoken language in the UAE is Arabic. In 2021, the EF English Proficiency Index demonstrated that the UAE is low proficient in English, with a ranking of 69 out of 112 countries [17]. Thus, KU CMHS medical students will likely provide care for Arabic speaking patients with limited English proficiency during their clinical rotations and residency training.

The Arabic program was offered to pre-clinical medical students in their first and second years of training. Clinical students in the third and fourth years were also eligible to attend when they were not on clinical rotations.

### Program description

In 2020, we launched a pilot year-long Arabic medical terminology and language program using a peer assisted learning (PAL) model. PAL is a peer teaching approach where students help each other to learn. Guided by an instructor, the students work in small groups and alternately take on the roles of tutor and tutee to teach and learn the information [18]. PAL programs that focus on peer mentorship and tutoring have been widely implemented in medical schools to support academic performance by providing a safe space for students to interact with peers and engage in active learning in an informal setting [18]. Studies have documented a wide range of benefits from incorporating PAL activities in the medical school curriculum, including enhancing leadership skills, boosting self confidence, and fostering community [19]. We believed that the PAL approach would create a comfortable environment to enable native Arabic speakers to practice medical terminology and history taking in Arabic, while teaching and supporting non-Arabic speakers to learn a foreign language.

The main objective of this pilot program was to teach the medical vocabulary needed to perform a history and physical examination, counsel patients on treatment plans, and provide medication instructions to Arabic speaking patients. Optional one hour classes were offered biweekly and taught by bilingual (Arabic and English) teaching staff. During the COVID-19 pandemic, virtual sessions were held. Topics included conversational Arabic, identifying body parts and organs, eliciting a chief complaint and medical history, providing instructions for taking medications, and providing lifestyle and behavioral counseling for disease management. Class design followed the Presentation - Practice - Production (PPP) methodology [20]. The application of PPP has been shown to facilitate students’ vocabulary mastery in a foreign language [20]. First, the presentation component included PowerPoint presentations and instructor-led discussions that introduced the day’s topic and vocabulary. This didactic component was followed by practice, in which Arabic and non-Arabic speaking students practiced the vocabulary together through the PAL approach. During the production phase, students used the newly learned vocabulary by participating in role-playing exercises or completing worksheets. Session objectives are listed in Table 1.

**Table 1:** Session objectives

1. Initiate social conversations in Arabic, such as greetings and inquiring about patient’s wellbeing
2. Recite days of the week in Arabic
3. Count from 1-20 in Arabic
4. Identify different body parts and organs in Arabic
5. Elicit general and system-specific chief complaints in Arabic
6. Elicit details of the chief complaint, such as frequency, severity, etc.
7. Perform a comprehensive patient history (medication history, past medical history, family history, history of present illness, etc.) in Arabic
8. Describe different drug formulations (ie tablet, syrup)
9. Communicate medication administration and frequency to patients in Arabic
10. Provide behavioral counseling (diet, exercise, smoking cessation, etc.) in Arabic

## Results

The pilot year-long Arabic medical terminology and language program was offered in the 2020-21 and 2021-22 academic years. Of 59 total students in the first 2 cohorts of students eligible to participate in the Arabic classes, the majority were women (*n* = 41; 69.5%) and 47 (79.7%) were native Arabic speakers. On average, 26 students (44%) attended each class (range 15–28; SD = 5.2). The majority of the non-Arabic speaking students (70.9%) attended most of the classes, and approximately 30% of the native Arabic speaking students also attended most classes, resulting in a 2:1 ratio of Arabic to non-Arabic speakers at sessions.

To assess perceptions and overall satisfaction with the classes and the program’s impact on the students’ communication skills during clinical encounters, an anonymous web-based survey (Additional file 1) was administered to these first two cohorts of students (*N* = 59) in July 2022. Forty-eight students (81.4%) completed the survey. Almost all of the survey respondents (44/48; 91.7%) attended at least half of the Arabic classes offered, even though they were optional. The vast majority of students (44/48; 91.7%) felt that the peer-teaching approach to Arabic language was an effective learning technique. Moreover, 43 students (89.6%) reported using the vocabulary learned in the Arabic classes during home visits and clinical rotations, and 42 students (87.5%) admitted that the classes made them feel more comfortable in their Arabic communication skills. There were no significant differences in perceptions between native and non-native Arabic speakers. The majority of students surveyed (41/48; 85.4%) were interested in attending more Arabic classes.

This study was deemed exempt from institutional review board review by the Khalifa University Research Ethics Committee [H22-030].

## Discussion

The pilot Arabic medical terminology and language program was well received by both native and non-native Arabic speakers and provided students with increased comfort and confidence in caring for the local Arabic speaking patient population. Students reported applying the lessons learned in class to help them better communicate with patients during their clinical interactions. Our findings suggest that the PAL framework can be effective in bridging the linguistic gap. This simple, easy to implement model can be adopted by medical schools worldwide.We learned many lessons during implementation of the pilot program. First, the peer teaching model was successful in engaging both native and non-native Arabic speakers and served the needs of both groups. Also, we realized that although the program is not a mandatory component of the curriculum, horizontal integration with other courses is necessary. The sessions offered should reinforce and support the topics covered in the organ systems courses and clinical skills sessions. This could also enable peer teaching of curricular material, while practicing Arabic language skills. Further, to optimize the practice and production phases, we have made available video resources of the topics prior to each session. These can also be reviewed after sessions for reinforcement and are available to students who are unable to attend. Finally, we are in the process of developing simulated patient clinical scenarios in Arabic to assess the impact of the program on student communication skills.

Limitations of this pilot study include the small number of students in a single institution. Also, surveys measured satisfaction with the teaching method and students’ perceived comfort levels and improvements in confidence. Objective measures, such as OSCEs or interactions with simulated patients, are needed to assess for changes in confidence or language skills after participation in the program. Despite these limitations, the program was feasible and low cost. Student participation was high- even within an already crowded medical school curriculum.

## Conclusion

Physician-patient language concordance can contribute to high quality patient care. A feasible, low cost program, using a peer teaching approach, can improve students’ comfort in communicating with patients in the local language, and can be adopted by medical schools worldwide to help address the challenge of language barriers in healthcare.

## Supplementary Information

**Additional file 1.** Survey Questions.

## Data Availability

The datasets generated and/or analysed during the current study are not publicly available to maintain student confidentiality but are available from the corresponding author on reasonable request.

## Abbreviations

PAL: Peer assisted learning
UAE: United Arab Emirates
