# Supplementary material for: Promoting physician-patient language concordance in undergraduate medical education: a peer assisted learning approach
Source: BMC Med Educ. 2023 Jan 3;23:1. doi: 10.1186/s12909-022-03986-4 (PMC9807417; doi:10.1186/s12909-022-03986-4)
Supplement: Supplementary file 1 — Additional file 1. Survey Questions. [file 12909_2022_3986_MOESM1_ESM.docx]

Appendix A- Survey Questions

1. Did you attend Arabic Classes
2. If no, why not?
3. How often did you attend Arabic classes?
4. Did you find practicing the Arabic language in class with your peers using the PAL approach an effective learning technique?
5. Did you use what you learned in your home visits and/or hospital rotations?
6. Did the classes make you feel more comfortable in your Arabic communication with patients?
7. Would you be interested in attending more Arabic classes?
